# Quantifying the Effects of Detraining on Female Basketball Players Using Physical Fitness Assessment Sensors

**DOI:** 10.3390/s25071967

**Published:** 2025-03-21

**Authors:** Enrique Flórez-Gil, Alejandro Vaquera, Daniele Conte, Alejandro Rodríguez-Fernández

**Affiliations:** 1 Faculty of Health Sciences, Universidad Isabel I, 09003 Burgos, Spain; enrique.florez@ui1.es; 2 VALFIS Research Group, Institute of Biomedicine (IBIOMED), Faculty of Sciences of Physical Activity and Sports, University of León, 24007 León, Spain; alrof@unileon.es; 3 School of Sport and Exercise Science, University of Worcester, Worcester WR2 6AJ, UK; 4 Department of Movement, Human and Health Sciences, University of Rome “Foro Italico”, 00135 Rome, Italy; daniele.conte@uniroma4.it

**Keywords:** training cessation, basketball, female, performance, technology, sensors

## Abstract

This study leverages physical fitness assessment sensors to investigate the effects of a brief in-season break (detraining period) on the physical performance of female basketball players. Sixty-seven players (Senior (n = 19), U18 (n = 19), and U14 (n = 29)) were evaluated before and after a 3-week break using sensor-derived data from a countermovement jump (CMJ), an Abalakov jump (ABK), a linear speed test (20 m sprint), a seated medicine ball throw test (SMBT), and a Basketball-Specific Agility Test (TEA-Basket). The Total Score of Athleticism (TSA), computed as the mean Z-Score across tests, served as a composite indicator of physical fitness. Data obtained from performance sensors revealed significant interactions between time and category for the CMJ, ABK, 20 m sprint, and SMBT, while TEA-Basket measurements showed no significant changes. Time and baseline fitness level interactions were also significant for the CMJ, ABK, and SMBT but not for sprint time or the TEA-Basket. Despite observed declines in explosive strength, speed, and upper-body power across all groups, TSA scores remained stable. These findings underscore the utility of sensor-based evaluation methods in highlighting the adverse effects of short-term detraining and emphasize the necessity of tailored training strategies during competitive breaks.

## 1. Introduction

Female basketball matches are characterized by players covering distances of 4404–7558 m and performing 23–44 movements per min [1]. Additionally, it has been documented that female players perform 652 ± 128 movements, one every 2.8 s, and spend 5.7% of their active playtime at high intensities [2]. Therefore, the ability to perform repeated sprints primarily characterized by short and linear bursts is considered a crucial factor [3] underlining the necessity to develop a high level of physical fitness to meet the match demands [4]. Furthermore, basketball is an open-skill sport that requires players to perform technical actions while making decisions based on the game context during these physical activities [5]. For this reason, the assessment of physical fitness should not be limited to traditional fitness tests but should also include game-specific demands.

While the physical tests have been assessed in female basketball players following various training programs to assess their effectiveness [6,7], limited information is available regarding the impact of unloading or detraining periods. This aspect is crucial since, during the season, teams constantly engage in both training and matches [5] but also in different periods characterized by a reduction or complete absence of competition and/or training sessions [8]. These periods usually occur both during the transition periods between seasons and during the competitive season [9]. In fact, the competitive basketball season is characterized by the absence of competition during special moments, such as the Christmas holiday break, or in the case of short-term injuries, during which a decrease or cessation of training stimulus can apply. In turn, this situation can lead to a decline or deterioration of the adaptations achieved through training and, therefore, a decrease in the athletes’ performance [10]. The performance decline due to absence or insufficient stimulus has been described by some authors as detraining, or the partial or total loss of training-induced adaptations in response to an insufficient training stimulus [9]. Detraining has then been classified based on the duration of cessation or inadequate stimulus as short-term (i.e., <than 4 weeks) and long-term (i.e., >than 4 weeks) detraining [9].

Previous studies have shown that three weeks of inactivity is enough to cause a significant reduction in vertical jump performance [11], aerobic endurance [12], and anaerobic performance [13] in team sports athletes. In short-term detraining or inactivity periods, reductions of 4–14% in VO_2_max have been reported, while strength production has not been as affected by this short-term period [9]. Previous studies on female team sports (i.e., soccer) athletes, have shown that a two-week period of inactivity during the competitive season leads to a decrease in the repeated sprint ability performance (mean time of eight sprints, *p* < 0.001 ES = 2.04) and CMJ performance (*p* = 0.009, ES = 1.39) [13]. Considering the documented negative effect of detraining on team sports athletes, monitoring the detrimental effects of detraining on physical fitness seems crucial in order to apply specific training strategies to prevent performance declines during critical times of the season. However, there is a lack of research specifically focusing on female basketball players, highlighting the need for studies on this population. Furthermore, previous research has often focused on single-team studies [14], underscoring the necessity for studies with larger datasets or different age categories to generalize results to the broader female basketball population.

Previous research also demonstrated that the effects of detraining are determined by age and gender [15], duration of the detraining [9], and previous training load and fitness level [16]. In this regard, Rodriguez Fernández et al. [16] found that fit players (≥27.3 km·h^−1^ in 30 m sprint test) showed a greater impairment in the repeated sprint ability test performance after a 2-week in-season break period compared to slower soccer players. However, this study only classified more and less fit players based on the sprint test, which measures a specific physical quality, while it is warranted to analyze whether the overall physical fitness influences the level of detraining. With the idea of providing a holistic physical fitness assessment, the Total Score of Athleticism (TSA) has been proposed [17]. This method is designed to establish a single value for the overall performance of an athlete within a team by consolidating the results of various physical performance tests into a sum of Z-Scores [18]. Silva et al. [19] found no significant differences in TSA scores after 3 weeks of small-sided games training (three vs. three and five vs. five), but both players with low and high TSA scores (below and above 0.0) showed significant improvements in their 30–15 IFT performance. However, to the authors’ knowledge, there are no studies evaluating the effects of detraining based on the TSA in female basketball players.

Therefore, the aim of this study was to analyze the effects of a period of absence of training and competition on the physical performance of female basketball players based on age category and fitness level.

## 2. Materials and Methods

### 2.1. Experimental Design 

This study was carried out during the in-season break (3 weeks during the Christmas break) of the 2023–2024 competitive season, during which players were not involved in any competitive games or training sessions (team or individual sessions). Specifically, players were asked to abstain from any type of physical training (other than daily life activities) during the in-season break. After the break, players were individually interviewed to evaluate their adherence to this requirement. Before and after this period, the players completed the same fitness tests, including a countermovement jump (CMJ), an Abalakov jump (ABK), a linear speed test (20 m sprint), a seated medicine ball throw test (SMBT), and a Basketball Agility Specific Test (TEA-Basket). Passive standing recovery periods of 45 s after the jumping test, 5 min after the sprint test, and 3 min after the throwing test were included to perform all tests in a single assessment session before and after the inactive period. The tests were performed in the following order: first the jumping tests, followed by the linear sprint test, then the SMBT, and finally the TEA-Basket. The data collection was divided according to the training groups with all measurements being taken between 16:00 and 21:00, starting with U14 and U18 up to the Seniors. Prior to each session, players undertook a standardized warm-up along with their usual pre-match activation routine. Players were required to wear their usual training uniforms and basketball shoes during the tests, performed in their respective habitual training venues. Players were familiarized with the testing procedures (i.e., players made two submaximal attempts for each fitness test) the week before the commencement of the study.

### 2.2. Subjects

Sixty-seven female basketball players from three different teams (Senior (n = 19), U18 (n = 19), and U14 (n = 29)), all belonging to the same club, volunteered to participate in the study (Table 1). The Senior team was in the third division of the Spanish league for the second consecutive year (Tier 3) [20]. The U18 and U14 teams competed at the national level (Tier 2) [20]. All players had a minimum of three years of competitive basketball experience. Additionally, the participating players met the established inclusion criteria, which included the following: (a) having participated in at least regional-level competitions, (b) no injuries reported in the two months prior to the study, (c) completing all performance tests before and after the training cessation period, and (d) adhering to the cessation of activity during the experimental phase. Before the study began, all participants were informed about the benefits and risks, and each provided their informed consent. The study design received approval from the Ethics Committee of the University of León (Code: ETICA-ULE-004-2021).

### 2.3. Procedures

Jump height was measured using the MyJump 2.0 app (validity: r = 0.995 correlated with a force platform, *p* < 0.001; reliability: intraclass correlation coefficient = 0.997, *p* < 0.001) [21]. Each jump was recorded at 240 Hz with an iPhone XS mobile device (Apple Inc.; Cupertino, CA, USA). The evaluator was always recording from the same position and with the same distance from the participants (1.5 m) as the standard calibration according to the manufactory instructions. The players performed two countermovement vertical jumps, with hands placed on hips, with 45 s of passive standing recovery between attempts. Successively, players performed two ABK jumps, with natural arm swing, with 45 s of passive standing recovery between attempts. For all jump tests, there were no restrictions on the knee angle during the eccentric phase of the jumps, but players were required to keep their legs straight during the flight phase [22]. The highest jump height among the three attempts was used for the subsequent analyses. Overall, the tested players showed coefficients of variation (CV) of 15.4% and 17.6% for CMJ and ABK, respectively.

A photoelectric cell (Polifermo Light Radio, Microgate; Bolzano, Italy) encompassing a transmitter placed in front of a reflector that returns the signal were used to assess the 20 m sprint test. The transmitter and the reflectors were placed on tripods at the height of the hips (approximately 90 cm height) while being used to time the sprint tests. These devices were switched on just before the different tests were carried out, synchronized, and checked to ensure their correct functioning before the first participant carried out the test. This technology has been previously considered as a reference tool in time measurement and is recommended for recording accurate and reliable results (0.03 s of standard error of measurement and CV= ~2%) in scientific research [21,23].

To test players’ agility, the traffic light Witty SEM© (Microgate, Bolzano, Italy) consisting of a matrix of 7 × 5 LEDs showing different symbols and colors, by means of which the athletes can interact thanks to their proximity sensors, was used. These traffic lights can have different methods of activation, after a photocell barrier, so that when crossing the barrier, the traffic light will be activated automatically. The traffic light indicates the required signal (random or not) when crossing the barrier or independently acting as proximity photocells. This device is inserted on top of a tripod which is about 90 cm above the ground, where the player can perfectly see the command sent by the device. The tests with the traffic lights, both quantitatively and qualitatively, were developed using a display, with which we can choose the reaction time of the traffic lights and the shapes and colors that we want to appear on the screen, although they will always appear randomly [24]. The sprint test consisted of three 20 m sprints interspersed by 180 s of passive recovery [25]. The best attempt of the three was selected for further analysis. The players began each run 0.5 m from the starting line (first photoelectric cell), and times were recorded at 5 m and 10 m split, and over the total 20 m distance. The CV for the sprint test in the current sample of players was 5.8%, 5.6%, and 7.6% for the 20, 10, and 5 m split, respectively.

**Figure 1:**
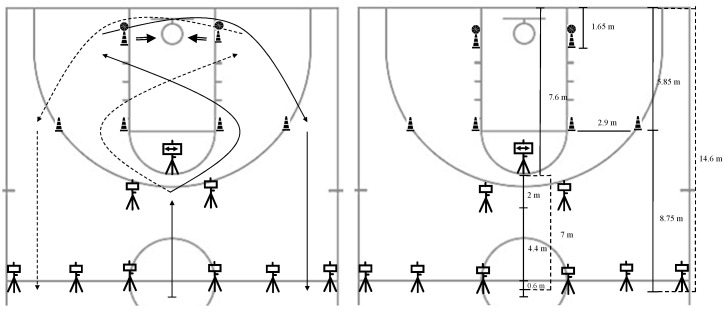
The Basketball-Specific Agility Test scheme.

The Basketball-Specific Agility Test (TEA-Basket) (Figure 1) was used to assess reactive agility. The players had to complete the test circuit as quickly as possible. They started the test 0.5 m from the starting line and performed two attempts with 60 s of passive rest in between, using the fastest attempt for analysis. Performance time was measured using a photoelectric cell, used in previous agility tests on basketball players [22]. The CV for the Basketball-Specific Agility Test in the current sample of players was 11.05%.

Upper body strength was assessed using the SMBT. Players held a 2 kg medicine ball in their hands while seated with their backs against the wall, with their upper limbs abducted and elbows flexed. They were then instructed to throw the medicine ball straight ahead as far as possible using a basketball chest pass without losing the contact of their head, shoulders, and back with the wall. Two throws were performed with 2 min of passive recovery between them [26]. A 10 m tape measure was placed on the floor with one end fixed to the wall. The medicine ball was covered with magnesium carbonate (gym chalk) to leave a clear mark on the floor after each throw to easily determine the distance reached. The total distance thrown was measured and used as the performance indicator of the test. The CV for the SMBT in the current sample of players was 24.0%.

### 2.4. Statistical Analysis

Descriptive statistics were calculated as mean ± standard deviation (SD). Moreover, the change in tests performance is presented as a percentage (Δ = (([after value − before value]/before value) × 100).To analyze the differences among fit and unfit players, the median split technique was used to divide the pooled participants of the entire team in each of the tests scores before the detraining period [16]. After confirming the normality of the analyzed variables using the Shapiro–Wilk test, differences between age groups and changes at different timepoints were examined using two-way repeated measures analysis of variance (ANOVA) (time [pre- vs. post-detraining] × category [U14, U18, and Senior]). In addition, the same procedure was used to test the interaction of time (pre- vs. post-detraining) × baseline level (fit vs. unfit) for the variations in CMJ, ABK, TEA-Basket, SMBT, and TSA. Bonferroni post hoc tests were used to locate any significant main effects between pairwise comparisons. Partial eta-squared (η_p_^2^) was determined to quantify the effect size (ES) and were interpreted as follows: small (<0.06), moderate (0.06–0.13), and large (>0.14) [27]. Additionally, Cohen’s d (using pooled standard deviation) was used to analyze the standardized effect size of pairwise comparisons [28,29]. The CV was determined as (SD × mean^−1^) × 100 to assess the variability of each test [28]. All analyses were performed using SPSS software (version 26.0, SPSS Inc.; Chicago, IL, USA). Statistical significance was accepted at *p* < 0.05. Once the performance values were established, the TSA was calculated as the average of the Z-Score obtained by the player in the CMJ and ABK tests and the value obtained in the 20 m sprint, SMBT, and the TEA-Basket according to the following formulas:

$$Zscore=\frac{player\;test\;result-mean}{Standard\;deviation}$$

$$TSA=\frac{Zscore\;CMJ+Zscore\;ABK+Zscore\;20m+Zscore\;SMBT+Zscore\;TEA\_Basket}{5}$$

## 3. Results

The repeated measures ANOVA revealed a significant interaction (time × category) in the CMJ (F = 8.411; *p* = 0.001; η_p_^2^ = 0.279, large), ABK (F = 7.420; *p* = 0.001; η_p_^2^ = 0.188, large), 20 m sprint time (F = 19.824; *p* < 0.001; η_p_^2^ = 0.236, large), and SMBT (F = 3.304; *p* = 0.043; η_p_^2^ = 0.094, moderate) but not in the TEA-Basket (F = 0.616; *p* = 0.543; η_p_^2^ = 0.019, small). The Senior and U18 players showed a significantly higher performance than the U14 players before the detraining period in all the fitness tests (Table 2). Additionally, there was a significant impairment in performance after the detraining period in all categories and test, except in the agility test. It should also be noted that there was an increase in body weight during the cessations of activity, which may, to some extent, have affected the decrease in performance that can be observed in the physical fitness test.

The repeated measures ANOVA revealed a significant interaction (time × baseline level) in the CMJ (F = 22.582; *p* < 0.001; η_p_^2^ = 0.258, large), ABK (F = 9.822; *p* = 0.005; η_p_^2^ = 0.117, moderate), and SMBT (F = 2.897; *p* = 0.039; η_p_^2^ = 0.093, moderate) but not in the 20 m sprint time (F = 1.941; *p* = 0.168; η_p_^2^ = 0.029, small) and TEA-Basket (F = 1.186; *p* = 0.312; η_p_^2^ = 0.036, moderate) (Table 3).

**Figure 2:**
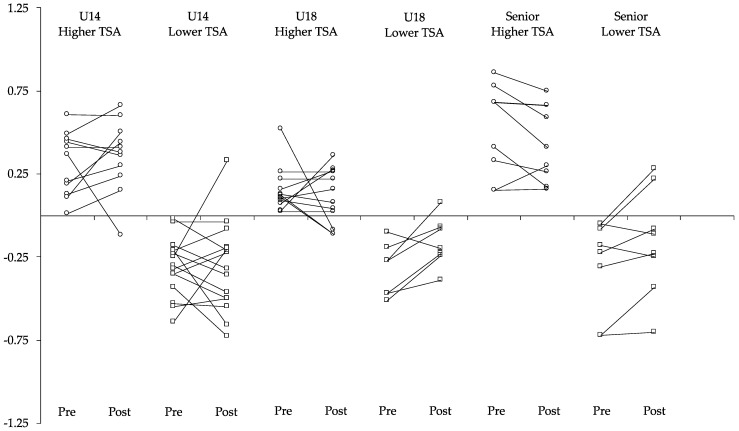
Physical fitness variations in players with lower and higher TSA levels. TSA = total score of athleticisms; Pre = pre-detraining assessment; Post = post-detraining assessment; Circle = players with high TSA; Square = players with low TSA; the lines mean intra-individual variation in each player.

The repeated measures ANOVA revealed no significant interaction for time (pre- vs. post-detraining) and fitness level (lower TSA vs. higher TSA) in U14 (F = 0.030; *p* = 0.863; η_p_^2^ = 0.001, small), U18 (F = 1.384; *p* = 0.256; η_p_^2^ = 0.075, moderate)*,* and Senior players (F = 3.602; *p* = 0.075; η_p_^2^ = 0.175, large) (Figure 2).

## 4. Discussion

The main aims of this study were to analyze the effects of a period of absence of training and competition (3-week in-season break) on physical performance, and to determine if the category or fitness level can modulate the detraining effects in female basketball players using sensor-based evaluation methods. The major findings of this study were that a 3-week period of inactivity is sufficient to decrease jump capacity, sprint performance, and medicine ball throw ability, but it does not affect agility time, regardless of the category or initial fitness level in female basketball players. Based on these results, coaches and strength and conditioning coaches should consider these findings when planning training activities during rest periods (i.e., in-season and off-season) and in training sessions following periods of inactivity. This is crucial because the decrease in strength and neuromuscular control has been associated with a higher risk of injuries, especially in females participating in sports such as basketball or soccer [30].

To the best of our knowledge, this is the first study showing the response of physical performance after an in-season break in female basketball players. A previous study [13] analyzed the effects of a specific break period (2-week in-season break) on RSA (Repeated Sprint Ability), countermovement jump (CMJ), and locomotor performance in SSGs in young female soccer players, showing a decrease in the mean time of the repeated sprint ability test (*p* < 0.001, effect size = 2.04) and countermovement jump (*p* = 0.009, effect size = 1.39) performance but not in the best time of repeated sprint ability. However, the authors did not control the category and fitness level previous to the break. It is possible that the category (i.e., age) and physical fitness level impact detraining effects [16].

Our results revealed a significant decrease in the jump capacity (i.e., CMJ and ABK), 20 m sprint time, and SMBT from pre- to post-detraining across all categories except for the ABK in U14 players. In a similarly designed study, Rodriguez Fernández et al. [31] showed a greater decline in performance in an RSA test after a period of inactivity in professional players compared to younger soccer players, suggesting biological conditioning factors and different levels of physical fitness as possible reasons [32,33]. In contrast, our results indicate a detrimental effect of the detraining period on physical fitness regardless of the players’ category. A possible explanation for our results could be that our participants possess lower fitness levels. In fact, previous studies have reported better CMJ (22–48 cm) [34] and 20 m sprint (3.27–3.46 s) [35] values for female basketball players. Consequently, the lower fitness levels may have led to the lack of a differential effect based on category. It was expected that Senior and U18 players would have a higher fitness status, which might have modulated the detraining effect based on category, as fitter individuals tend to lose more performance compared to those who are less fit from the beginning.

In our study, the detraining period significantly decreased the jump height in the CMJ (4.9 ± 4.6%). Previous research has corroborated that a short cessation period causes a 11.7.2% decline in CMJ height in young healthy boys between 10 and 13 years [36] and 15.4% in vertical jump height in female basketball players after 3 weeks [14]. Therefore, to maintain the ability to produce force in the lower body, female basketball players need to continue training. This influence of detraining on the CMJ is also observed in other team sports such as rugby, with a decrease of 0.94% [37], although this CMJ was restored in a short period of time, less than two weeks [38]. Moreover, regarding tests related to neuromuscular capacity (i.e., 20 m sprint time) for a three-week activity cessation, we report decreases of 2.2 ± 3.80%, while other studies report decreases of 1.9 ± 6.7% [39]. However, there are studies that obtain an improvement in performance after detraining or the cessation of activity. This may be due, among other factors, to the taper effect [40]. Santos and Janeira (2011) [41] observed an improvement in performance in adolescent female basketball players after a four-week training cessation period. In this study, the players increased their values in the CMJ (1.05%), SJ (6.3%) and upper body strength tests (0.72%). Although, this effect usually occurs in players who have a high training volume and/or poor load management [42]. Since the intensity of previous training can condition the effects of tapering or the cessation of activity, it is possible that those players underwent the tapering process and thus improved their marks [40].

According to Suchomel et al. (2016) [43], sprinting is a complex skill that demands high levels of muscular output, power, and technique. Earlier research indicates that sprinting is a specialized talent that requires training, particularly at maximal or near-maximal levels, and genetics [44]. Inadequate stimulation may arise from failing to meet these benchmarks, which could lead to performance reductions [45]. Our results showed a decrease in 20 m sprint times across all categories. Previously, Rodríguez Fernández et al. [31] reported a significant impairment (*p* = 0.008; ES = 1.66) in best times in RSA tests in female soccer players after an in-season break period (2 week). Similarly, male soccer players have shown a significant decrease in RSA (total time spent on the test: 33.41 + 0.96 s vs. 34.11 + 0.92 s; *p* < 0.01), as well as in slow-twitch fiber fraction (56 + 18% vs. 47 + 15%; *p* < 0.05) and VO^2^ kinetics (*p* < 0.05) after a short-term detraining period [46]. Therefore, even a short period of inactivity is sufficient to impact neuromuscular performance and reduce sprint performance [16].

Our results indicate a significant decrease in performance for both fit and unfit players (i.e., those above or below the average). However, fit players exhibit a more marked decline compared to unfit players (1.5–6.8%, ES = 0.31–0.83 vs. 0.7–5.9%, ES = 0.26–0.47, respectively). Due to their greater initial performance level, fit players may have experienced more pronounced detraining effects, with increased negative impacts on fast-twitch muscle fibers, the ability to use ATP and phosphocreatine, and a greater production of metabolic by-products [9,47,48,49]. In summary, regardless of age, fit players appear to experience a more pronounced decline in their performance following periods of detraining compared to their unfit counterparts. Consequently, fit team sport athletes may require greater attention from technical staff, as they are more prone to losing their physical fitness abilities during various stages of preparation. This is crucial because the decrease in strength and neuromuscular control has been associated with a higher risk of injuries, especially in women who practice sports like basketball or soccer [30].

Agility has been defined as a rapid whole-body movement involving the change in velocity or direction in response to a stimulus, encompassing two main components: change in direction and speed and perceptual and decision-making factors [50,51]. Therefore, the tests used for its evaluation must include both aspects [52]. To our knowledge, this is the first study that analyzes the effects of detraining in tests that incorporate changes in direction, response to a stimulus, and specific actions. No decline in performance was observed in the TEA-Basket, regardless of category or initial fitness level. These effects could be due to three reasons: either this short period of inactivity does not impact agility, or the designed test is not sensitive enough to detect changes caused by detraining; additionally, this test involves both physical and reaction components, and maybe the reaction components are not impacted by the detraining period. Future studies are needed in this area.

The Total Score of Athleticism (TSA) has been proposed as a method to provide a single, holistic fitness score [17]. Additionally, soccer players with both high (TSA > 0) and low (TSA < 0) TSAs did not show changes in their TSA values after three weeks of training through small-sided games [19]. Although previous studies have examined the effects of detraining in team sports participants [16,41,45], as far as we know, no study has analyzed the effects of detraining in female basketball players according to the TSA. These outcomes may be due to several factors. It is possible that if the TSA is not sensitive to adaptations resulting from training [19], it might also be insensitive to the effects of detraining. Moreover, the TSA calculation was based on only four measures; none of which included the aerobic component. While the different tests individually showed a decline in performance, the combined evaluation using the TSA might have masked these individual declines.

This study is not without limitations. First, the TSA measurement did not include an aerobic test, which could have provided a more comprehensive assessment of fitness changes. Future studies that include tests such as the Yo-Yo Intermittent Test or the 30–15 Intermittent Fitness Test could clarify this situation. Second, this study only covered a short detraining period; future studies that include longer periods might show different results. Additionally, examining psychological and motivational factors during detraining could provide a more holistic understanding of performance changes.

## 5. Conclusions and Practical Applications

A short period (3-week in-season break) of inactivity appears to negatively impact physical fitness (CMJ, ABK, 20 m sprint time, and SMBT) in female basketball players, regardless of their category or initial fitness level. However, agility performance, as measured by the TEA-Basket, remained unaffected. The TSA did not change significantly after the detraining period. Therefore, in the event of an in-season break, training is necessary to provide the minimum stimuli to maintain the players’ fitness, which allows them to continue training or competing. Coaches should consider these findings when planning training schedules after an in-season break to recover the performance metrics lost during the break and prevent injury risks. With these findings, future lines of research should look at extending this period of cessation of activity, such as off-season breaks, to determine whether the magnitude and rate of recovery varies with longer periods of detraining. In addition, we may also consider incorporating other assessments, such as aerobic testing or cognitive performance measures, to provide a more complete assessment of the athlete. Finally, it would be very interesting to include an individualized analysis of the players’ fitness level in relation to their peak performance.

## Figures and Tables

**Table 1:** The descriptive characteristics of the female basketball players.

Characteristics	Senior (n = 19)	U18 (n = 19)	U14 (n = 29)	Total (n = 67)
Age (years)	20.2 ± 1.8	16.4 ± 0.8	13.4 ± 0.5	16.2 ± 3.0
Height (cm)	173.7 ± 8.0	169.5 ± 7.1	162.0 ± 7.0	167.4 ± 8.8
Wingspan (cm)	173.8 ± 11.1	167.7 ± 7.2	159.8 ± 8.6	166.0 ± 10.7
Weight Pre (kg)	73.6 ± 9.4	63.1 ± 8.4	51.6 ± 9.1	61.1 ± 12.8
Weight Post (kg)	74.3 ± 9.2	64.3 ± 8.4	52.5 ± 9.5	62.0 ± 12.9

U18 = under 18 years; U14 = under 14 years.

**Table 2:** Physical fitness test outcomes (mean ± standard deviation) pre- and post-detraining (3-week in-season break) in female basketball players according to age group.

	Age Group	Pre-Detraining (Mean ± SD)	Post-Detraining (Mean ± SD)	Comparison Between Timepoints		
				Δ (%)	*p*	ES, *Magnitude*
CMJ height (cm)	Senior	25.91 ± 3.02 ^a^	23.96 ± 3.26 ^a^	−7.6 ± 5.5	**0.001**	0.64 *moderate*
	U18	24.20 ± 3.95 ^a^	22.93 ± 3.67 ^a^	−5.0 ± 4.2	**<0.001**	0.34 *small*
	U14	21.06 ± 2.29	20.38 ± 1.99	−3.1 ± 3.2	**<0.001**	0.32 *small*
	Total	23.33 ± 3.65	22.12 ± 3.28	−4.9 ± 4.6	**<0.001**	0.35 *small*
ABK height (cm)	Senior	30.75 ± 4.34 ^a,b^	29.44 ± 4.10 ^a,b^	−4.1 ± 5.5	**<0.001**	0.32 *small*
	U18	27.45 ± 4.28 ^a^	25.47 ± 4.36 ^a^	−7.1 ± 7.4	**<0.001**	0.47 *moderate*
	U14	23.19 ± 2.42	22.83 ± 2.23	−1.5 ± 1.9	0.194	0.16 *small*
	Total	26.54 ± 4.75	25.45 ± 4.41	−3.8 ± 5.5	**<0.001**	0.24 *small*
20 m sprint (s)	Senior	3.53 ± 0.20 ^a^	3.60 ± 0.26 ^a^	−2.0 ± 4.9	**0.033**	0.31 *small*
	U18	3.66 ± 0.22	3.73 ± 0.20	−2.1 ± 4.5	**0.026**	0.34, *small*
	U14	3.72 ± 0.15	3.81 ± 0.16	−2.4 ± 2.3	**0.001**	0.59, *moderate*
	Total	3.65 ± 0.20	3.73 ± 0.22	−2.2 ± 3.8	**<0.001**	0.38, *small*
SMBT (m)	Senior	4.04± 0.36 ^a,b^	3.72 ± 0.43 ^a,b^	−7.9 ± 6.4	**<0.001**	0.83, *large*
	U18	3.34 ± 0.39 ^a^	3.25 ± 0.32 ^a^	−2.2 ± 7.8	**<0.001**	0.26, *small*
	U14	2.57 ± 0.51	2.36 ± 0.48	−4.9 ± 4.8	**<0.001**	0.43, *moderate*
	Total	3.20 ± 0.76	3.00 ± 0.72	−6.0 ± 9.7	**<0.001**	0.27, *small*
TEA-Basket (s)	Senior	10.73 ± 0.66 ^a^	11.10 ± 0.64	−3.5 ± 3.2	0.236	0.58, *moderate*
	U18	11.32 ± 1.11	11.81 ± 1.04	−4.5 ± 3.6	0.121	0.47, *moderate*
	U14	11.79 ± 0.73	12.02 ± 0.81	−3.7 ± 6.4	0.781	0.31, *small*
	Total	11.36 ± 0.94	11.8 ± 0.92	−3.9 ± 4.9	0.071	0.48, *moderate*

CMJ = countermovement jump; ABK = Abalakov jump; SMBT = seated medicine ball throw test; TEA-Basket = Basketball-Specific Agility Test; U18 = under 18 years players; U14 = under 14 years; Δ = percentage change between pre- and post-detraining; ES = effect size; ^a^ = significant differences to U14; ^b^ = significant differences to U18; and bolded *p*-value denotes significant difference between timepoints. Significant level *p* < 0.05.

**Table 3:** Physical fitness test outcomes (mean ± standard deviation) pre and post detraining (3-week in-season break) in female basketball players according to baseline level.

	Fitness Group	Pre-Detraining (Mean ± SD)	Post-Detraining (Mean ± SD)	Comparison Between Timepoints		
				Δ (%)	*p*	ES, *Magnitude*
CMJ height (cm)	Fit (>23.03 cm)	26.24 ± 2.33 ^a^	25.85 ± 2.07 ^a^	−1.5 ± −11.0	**0.001**	0.64, *moderate*
	Unfit (≤23.03 cm)	20.32 ± 1.91	20.24 ± 1.74	−0.7 ± −9.2	**<0.001**	0.34, *small*
ABK height (cm)	Fit (>25.71 cm)	30.46 ± 3.38 ^a^	29.82 ± 2.89 ^a^	−2.21 ± −4.7	**<0.001**	0.32, *small*
	Unfit (≤25.71 cm)	22.74 ± 1.92	22.32 ± 1.94	−1.8 ± 1.0	**<0.001**	0.47, *moderate*
20 m sprint (s)	Fit (<3.63 s)	3.49 ± 0.12 ^a^	3.59 ± 0.16 ^a^	−2.75 ± −3.2	**0.033**	0.31, *small*
	Unfit (≥3.63 s)	3.79 ± 0.14	3.87 ± 0.17	−2.21 ± −1.9	**0.026**	0.34, *small*
SMBT (m)	Fit (>3.20 m)	3.85 ± 0.36 ^a^	3.59 ± 0.39 ^a^	−6.8 ± 6.7	**<0.001**	0.83, *large*
	Unfit (≤3.20 m)	2.58 ± 0.45	2.42 ± 0.47	−5.9 ± 4.3	**<0.001**	0.26, *small*
TEA-Basket (s)	Fit (<11.24 s)	10.58 ± 0.41 ^a^	11.1 ± 0.71 ^a^	−4.8 ± 2.8	0.236	0.58, *moderate*
	Unfit (≥11.24 s)	12.07 ± 0.67	12.39 ± 0.66	−2.6 ± 2.7	0.121	0.47, *moderate*

CMJ = countermovement jump; ABK = Abalakov jump; SMBT = seated medicine ball throw test; TEA-Basket = Basketball-Specific Agility Test; Fit = player above the mean; Unfit = players below the mean; Δ = percentage change between pre- and post-detraining; ES = effect size; ^a^ = significant differences to unfit. Bolded *p*-value denotes significant difference between timepoints. Significant level *p* < 0.05.

## Data Availability

The data presented in this study are available upon request from the corresponding author. The data are not publicly available due to the Organic Law 3/2018, of 5 December, on the Protection of Personal Data and Guarantee of Digital Rights of the Government of Spain, which requires that this information must be in custody.
